# Peripheral giant cell granuloma: a case series and brief review

**DOI:** 10.1308/rcsann.2023.0021

**Published:** 2023-06-27

**Authors:** S Fligelstone, D Ashworth

**Affiliations:** Swansea Bay University Health Board, UK

**Keywords:** Epulis, Report, Features, Management, Recurrence

## Abstract

We report three varied presentations of peripheral giant cell granuloma and provide an up-to-date summary on the diagnosis, treatment and prognosis of this everyday swelling, including lessons learned.

## Background

Peripheral giant cell granuloma or giant cell epulis is the most common of the giant cell lesions.^1^ The aetiology is thought to be epithelial hyperplasia in reaction to irritant factors such as trauma or calculus. Clinically, the characteristic appearance is a red to blue mass that is variably ulcerated occurring anterior to the first molars.^2^ The differential diagnosis for these features is broad and consequential. Therefore, histopathology is required for diagnosis and management.

## Methods

A literature review was undertaken for an unbiased view of current knowledge on this topic. The database MEDLINE was searched in November 2021 and again in August 2022 using the terms and filter (peripheral giant cell granuloma OR giant cell epulis) AND (Medline[tiab] OR (systematic[tiab] AND review[tiab]) OR meta-analysis[ptyp] OR CDSR [so]). Of the 23 results, one recent review was eligible. This was supplemented with a reference check of the included article and a forward citation search using Google Scholar.

### Case 1

**Figure 1:**
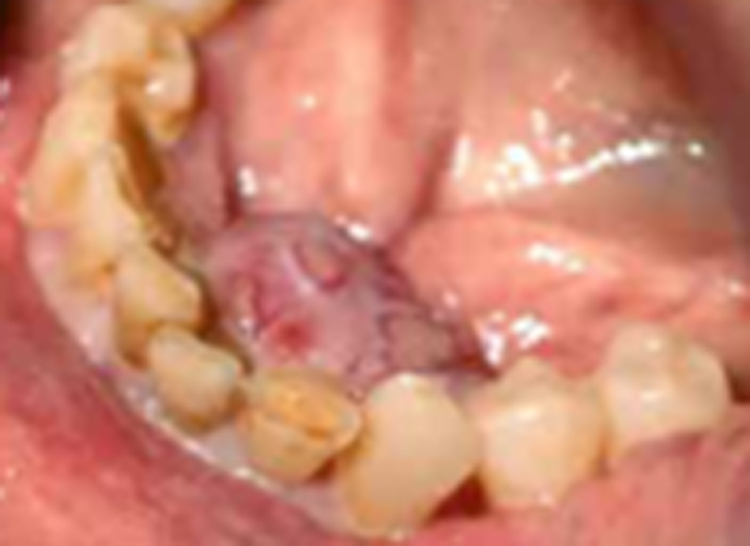
Broad-based ulcerated peripheral giant cell granuloma on the lingual gingiva of the anterior mandible

A 77-year-old male presented following an urgent suspected cancer (USC) referral with a 2-month history of a painless lump on the lingual gingiva of the lower anterior teeth. Medical and social background were unremarkable. Examination revealed a grossly neglected dentition with a 15mm lesion (Figure 1). Differential diagnoses were pyogenic granuloma, fibroepithelial polyp, giant cell granuloma (peripheral or central) and brown tumour of hyperparathyroidism. X-rays, biopsy and haematological investigations were undertaken. These established the diagnosis of peripheral giant cell granuloma with no bony involvement. Treatment was limited to excision and removal of calculus, the suspected causative factor. Interestingly, blood results displayed elevated alkaline phosphatase 141U/L (30–130U/L) and parathyroid hormone 7.1pmol/L (normal range 1.6–6.9pmol/L); however, this was later attributed to vitamin D deficiency. Final histology showed presence at the deep margin, with no recurrence evident at 6 months.

**Figure 2:**
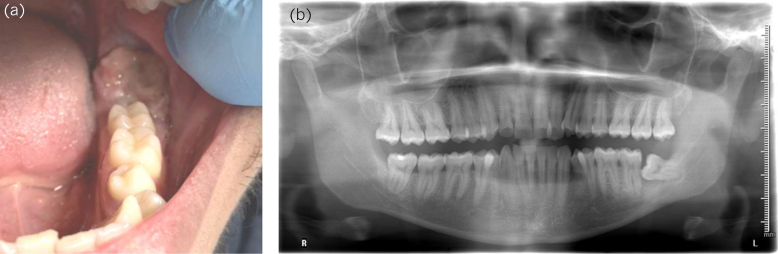
(a) Exophytic lesion on the alveolar mucosa of the right mandible. (b) Horizontally impacted lower left eight with associated bone loss but not resorption.

**Figure 3:**
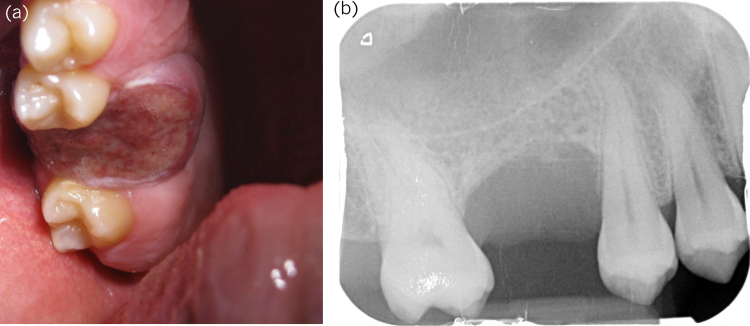
Peripheral giant cell granuloma: (a) manifesting as ulceration in the extraction socket of the upper right six; (b) periapical showing intact floor of sinus and absence of roots in area imaged.

### Case 2

A 35-year-old man attended via the USC pathway complaining of altered taste, bleeding and sore gingiva in the region of a horizontally impacted lower third molar. Medical history was clear although he smoked. On clinical assessment, an exophytic 22mm lesion was identified that the patient reported as having been present for 6–8 weeks (Figure 2a). Differentials were similar to Case 1; however, the site made squamous cell carcinoma and pericoronitis additional possibilities. After histological appearance confirmed giant cell lesion, excision along with surgical extraction of the associated wisdom tooth confirmed removal and complete resolution was seen at 6 months (Figure 2b). Underlying hyperparathyroidism was excluded.

### Case 3

Lastly, we consider a 24-year-old male reporting 4 weeks of pain, swelling and discoloration 1 year after extraction of an upper molar. This was again designated as USC. He was in good health and did not drink in excess. A 24mm ulcer occupied the socket with the margins rolled and indurated (Figure 3a). Localisation made it less likely to be peripheral or central giant cell granuloma, with more weight given to pyogenic granuloma, fibroepithelial polyp, brown tumour of hyperparathyroidism and squamous cell carcinoma (Figure 3b). A biopsy and bloods confirmed a peripheral giant cell granuloma, and parathyroid hormone of 8.4pmol/L (normal range 1.6–6.9pmol/L) and phosphate of 0.56mmol/L (normal range 0.8–1.5mmol/L) were fitting with mild primary hyperparathyroidism. A thyroid ultrasound was normal, with no convincing parathyroid adenoma visualised. Excision was achieved with the exception of one margin on microscopy and repeat bloods are pending.

## Discussion

Histologically, peripheral giant cell granuloma displays an unencapsulated proliferation of mononuclear and multinucleated giant cells in a vascular stroma.^3^ Comparing our series with Chrcanovic *et al*'s recent analysis of 2,824 cases, greater prevalence in the mandible is typical, contrary to a symptomatic male preponderance and absence of bone erosion; the latter accompanying almost one-third of their presentations.^4^ Therefore, special investigations should include radiography along with calcium, phosphate, alkaline phosphatase and parathyroid hormone levels. The vast majority of lesions are then excised surgically; however, with reported recurrence rates of 16%, the current study went on to explore prognosis and predictive factors. Probability was found to decrease with additional curettage (2.8%) or peripheral osteotomy (0%) and a combined approach concluded the first choice. Other modalities such as sclerotherapy, marginal resection and radiation were too few to compare. Another study featured two solely soft tissue resections using a carbon dioxide laser with no difference in postoperative course.^2^ Lack of removal of the periosteum was cited both in relation to a higher recurrence and being non-pathognomonic.^5^ Although partial excision was unintended in patients 1 and 3, it may suffice in these cases and others.

Common to reviews of pathological lesions, methodological issues mean that caution should be exercised in interpreting the discussed results for substantiation. Specifically, issues of scale prohibit individualised information to assess recurrence factors such as age, sex, location and so on, retrospective erroneous/incomplete data and follow-up to only 1 year mostly. The examples presented aim to provide as much detail as possible, illustrating both typical (Case 1) and atypical presentations. There remain poorly defined features of peripheral giant cell granuloma that require further research to aid in differentiation from other entities that bear a striking resemblance, even malignancies, and accurate management.

## Conclusions

Thorough clinical and radiological examination, biopsy and blood screen are the standard of care for these suspect lesions. Complete surgical removal and elimination of aetiological factors remain the literature-approved treatment.
